# Treatment of various degrees of white spot lesions using resin infiltration—in vitro study

**DOI:** 10.1186/s40510-018-0223-3

**Published:** 2018-08-06

**Authors:** Bassant A. Abbas, Eiman S. Marzouk, Abbas R. Zaher

**Affiliations:** Department of Orthodontics, Faculty of Dentistry, Alexandria University, Azarita, Alexandria, Egypt

**Keywords:** White spot lesions, Resin infiltration, Color change, Decalcification

## Abstract

**Background:**

This study was conducted to evaluate the efficiency of resin infiltration to improve the color of white spot lesions (WSLs) and to estimate the effect of different numbers of etching and resin infiltrant applications on the color change of WSLs with various depths. Ninety-six sound extracted premolars were subjected to acid attack inducing different depths of WSLs. Using a DIAGNOdent, teeth were divided into four main groups according to the depth of the WSLs: shallow enamel, deep enamel, shallow dentine, and deep dentine without cavitation. Then each of the main groups was subdivided into four groups: six teeth each with different protocols of resin infiltration as follows: 1 etching + 1 infiltrant application (EA), 1 etching + 2 infiltrant applications (EAA), 2 etchings + 1 infiltrant application (EEA), 2 etchings + 2 infiltrant applications (EEAA). Spectrophotometric analysis was measured at baseline (T0), after inducing the WSLs (T1), and following resin infiltration application (T2) for each group.

**Results:**

In shallow enamel, EA produced the least mean color difference (1.62 ± 0.85), with high significant difference (*P* < 0.001), when compared with the clinically detectable threshold (ΔE = 3.7). While in deep enamel, EAA showed the least mean color change (1.95 ± 0.4), with *P* < 0.001 when compared with the critical value. Also, in shallow dentine, the least mean change was noticed with EAA (3.0 ± 0.45), with *P* < 0.001 when compared with the clinical color detection threshold. Furthermore, in deep dentine, EAA had the least mean difference (3.76 ± 0.6) but with no significant difference, when compared with the clinically detectable threshold.

**Conclusions:**

As the WSL got deeper, the color of the lesion became more clinically visible. In shallow enamel, the best treatment option was one etching with one resin infiltrant application. For deep enamel and shallow dentine, one etching with two applications of infiltrant gave the best lesion masking. In deep dentine, it is advisable to perform one etching with two infiltration steps, taking in consideration that all deep dentine lesions without cavitation were partially masked, remained clinically detectable, and might require more invasive restorative procedures.

## Background

A beautiful smile, the most interactive communication skill of a person, is created with pleasing inherent proportions to one another and a pleasing tooth arrangement in harmony with the gums, lips, and face of the patient [1]. Early enamel decalcification, one of the most common side effects of fixed orthodontic treatment, manifests itself as white spot lesions (WSLs) [2, 3]. Initially, a WSL shows an apparently intact surface layer, followed underneath by the more porous lesion body, giving a chalky opaque appearance, as light is scattered mainly within the lesion body [4–7]. Scattering is caused at interfaces between substances with different refractive indices as enamel, water, and air [8]. Generally, WSLs could appear as early as 1 month after bracket placement; meanwhile, the formation of dental cavitation might require up to 6 months [9]. WSLs are frequently perceived on the dental buccal surfaces, around the brackets, mainly in the gingival area [10].

Prompt diagnosis and treatment are essential to avoid these lesions from turning into cavities [2]. The early detection of WSL is important as these lesions have the potential to be remineralized and monitored over time [11]. The DIAGNOdent 2190, known as DIAGNOdent Pen (DDPen, KaVo, Biberach, Germany), is a diagnostic device in which pulsed laser irradiation of a carious lesion causes the emission of reflected fluorescent light at 700–800 nm [12, 13]. Values between 10 and 25 indicate initial enamel carious lesion, values between 25 and 35 indicate superficial dentinal caries, and values over 35 indicate deep dentinal caries [14]. Several studies [15–17] evaluated its reliability and validity at quantification of smooth surface caries and WSLs.

Spectrophotometry aims to specify color by taking accurate measurements expressed either quantitatively or graphically. The Commission Internationale de l’Eclairage (CIE) developed a system that calculates the difference between two colors using a formula (∆E = [(∆L*)^2^ + (∆a*)^2^ + (∆b*)^2^]^1/2^, giving a single number as a value for color difference (∆E) [18]. Most studies set the proposed acceptance for color matching to be 3.7 units, above which the differences are clinically visible [19]. Previously in literature [20, 21], the spectrophotometer proved to have precise measurement and high accuracy.

Recently, with the advancing of materials, the infiltration concept was introduced in the field of dentistry. The purpose of the resin infiltration technique is to micro-invasively infiltrate the inter-crystalline spaces of enamel with polymerizable low viscous resin to arrest enamel lesions. Before a WSL can be infiltrated, it must be acid-etched to remove the hypermineralized pseudo-intact surface layer of enamel and thus permits resin infiltration into the body of the lesion [22, 23]. Since resin infiltration has a refractive index (1.48) that is comparable to that of enamel (1.65), thus, resin infiltration can completely mask the opaque color of less severe inactive WSLs and partially mask the appearance of moderate to severe WSLs [24].

In the 70s, Robinson et al. [25] investigated the concept of infiltrating a carious lesion with resorcinol-formaldehyde resin. The authors detected that in later experiments, the penetration of the resin was improved when the tooth surface was first etched with hydrochloric acid for 5–10 s. Gray and Shellis [26] concluded that as the time of etching and number of applications of resin increased, the percentage penetration of the resin material also increased. To clinically improve esthetic outcomes, Knösel and associates [27] recommended etching time of more than 120 s in WSLs that have been existent for a longer period of time and deeper lesions with a thicker intact surface layer. Arnold et al. [28] concluded that repeated conditioning with 15% hydrochloric acid resulted in reduction of surface roughness and increased the depth of the etched surface erosion. Nevertheless, the total erosion depth is rather shallow and hence negligible*.* Neuhaus and coworkers [29] showed that surface conditioning with hydrochloric acid proved to be superior for the penetration of the infiltrant, while etching with phosphoric acid only penetrates superficially into non-cavitated fissure caries lesion.

Meyer-Lueckel and Paris [30] and Liu et al. [31] showed that resin infiltration is capable of penetrating almost completely into caries lesions. Kim et al. [32] noted that the lesion depth and shade improvement were correlated. It was concluded that lesions deeper than the infiltration capacity of resin infiltrants might demonstrate insufficient esthetic improvement. Ou et al. [33] examined the treatment effect of resin infiltration on different degrees of enamel demineralization. The results indicated that treatment of high and low demineralization of enamel had a similar masking effect.

Up to our knowledge, this study was the first in the literature to evaluate the effect of different numbers of etching and resin infiltrant applications on the color change of WSLs with various depths. The null hypothesis was that different depths of WSL will have the same response of color change when using different times of etching and infiltrant applications.

## Methods

A sample size of 96 human premolars, with six teeth per group, was required to estimate clinical average change of color at various WSL depths, with a power of 80% and *α* = 0.05 using G-power software [34, 35].

Ninety-six human premolars extracted for orthodontic purpose were collected from subjects treated in the Department of Orthodontics, Faculty of Dentistry, Alexandria University. Teeth were divided into four main groups according to the depth of the induced WSL: shallow enamel (SE), deep enamel (DE), shallow dentine (SD), and deep dentine (DD) without cavitation. Then each of the main groups was further subdivided into four groups, six teeth each according to the resin infiltration protocol applied. The different infiltration protocols used were one etching with one infiltrant application (EA), one etching with two infiltrant applications (EAA), two etchings with one infiltrant application (EEA), and two etchings with two infiltrant applications (EEAA).

Criteria for selection: 1—sound teeth free from caries, 2—intact buccal surface with no visible cracks, stains, or hypoplastic areas, 3—no pretreatment with chemical agents.

### Teeth preparation

Following extraction, all teeth were cleaned with tap water and stored in artificial saliva solution (20 mmol/l NaHCO_3_, 3 mmol/l NaH_2_PO_4_, and 1 mmol/l CaCl_2_) at 37 °C and pH 7 to simulate the oral environment [36]. The solution was changed every day.

**Fig. 1:**
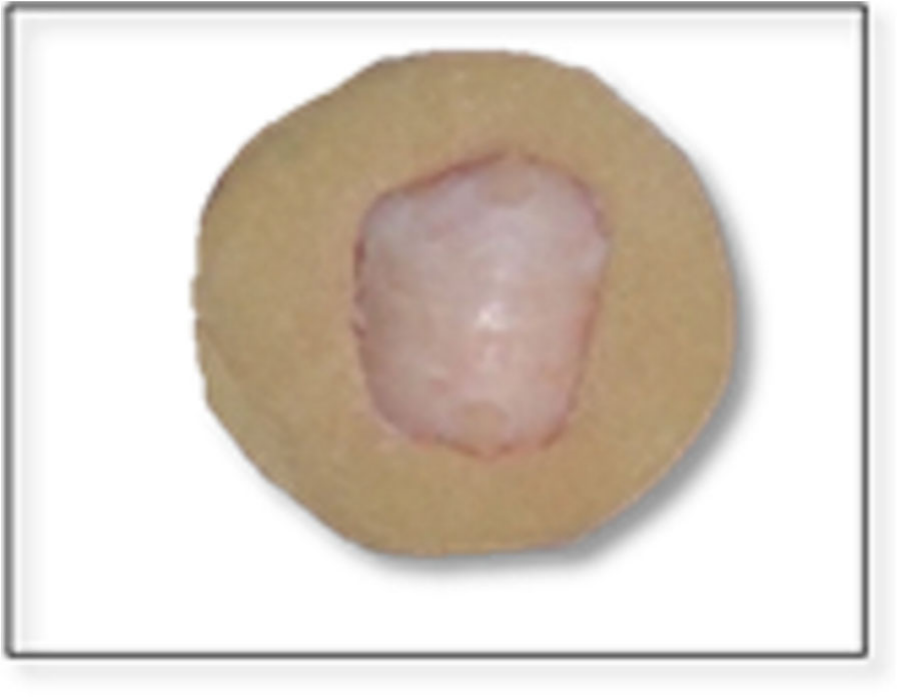
Tooth embedded in acrylic resin with the buccal surface parallel with the base

**Fig. 2:**
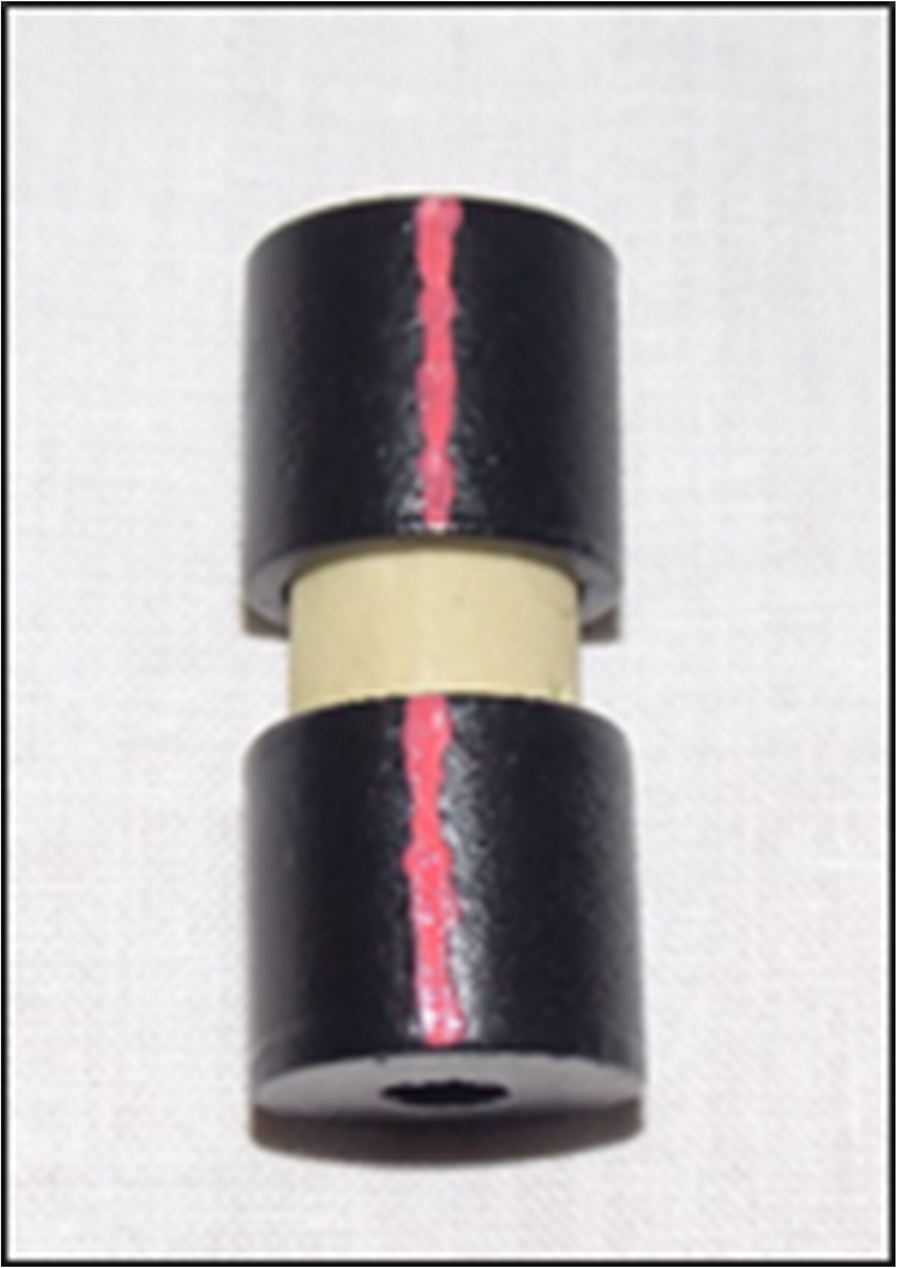
The placement of the specimen in the metallic mold

**Fig. 3:**
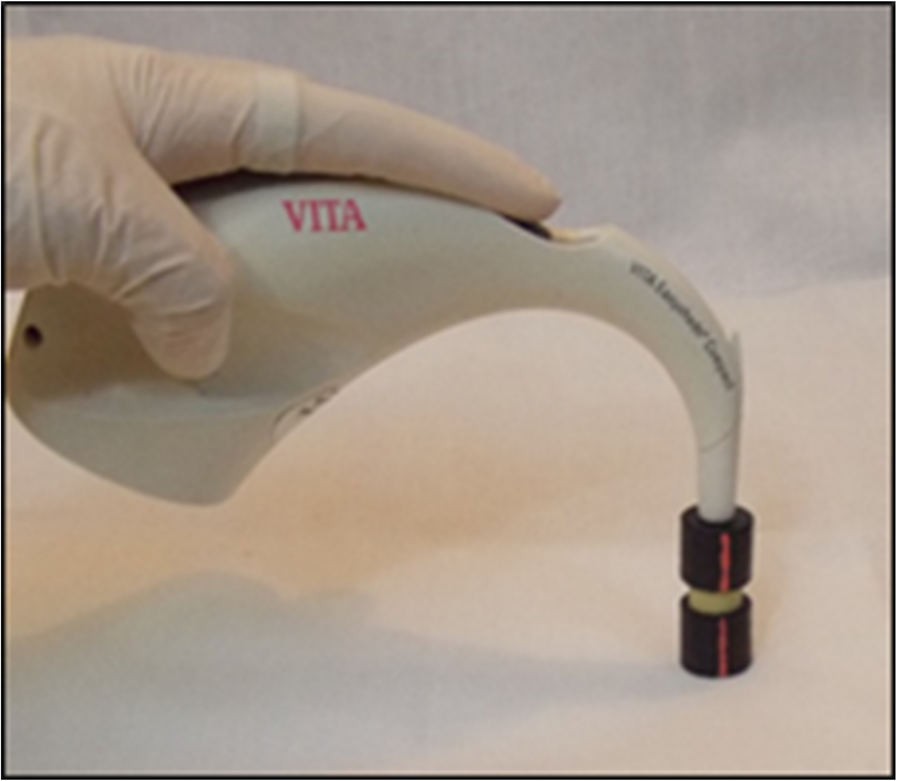
Spectrophotometer engaged in the mold ready for color measurement

**Fig. 4:**
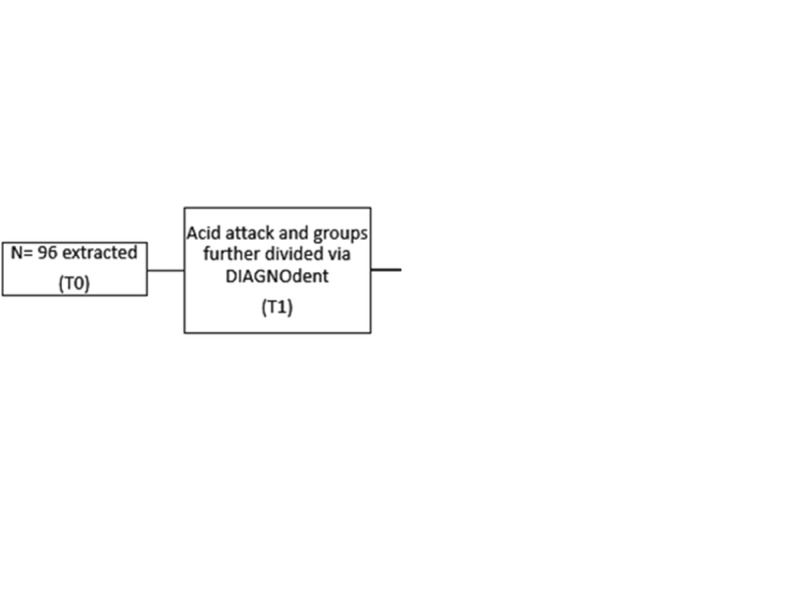
Flowchart of the study. T0, color of the teeth spectrophotometry measured at baseline. T1, color of the teeth spectrophotometry measured after the WSL formation. T2, color of the teeth spectrophotometry measured after different protocols of resin infiltration

1. At the beginning of the study, all teeth roots were removed at the cemento-enamel junction with a diamond disc.
2. In order to facilitate examination of the buccal surface, the lingual surfaces of the teeth were embedded down in self-curing acrylic resin held by a metal mold so that the buccal surface aligned parallel with the base of the mold (Fig. 1).
3. Prior to color measurement, all teeth were polished with rubber prophylaxis cup at a low-speed handpiece with a mixture of non-fluoridated oil-free pumice and water and then rinsed with running water [37].
4. A black painted metallic mold was fabricated to help hold the teeth in a standard position during color measurement (Fig. 2). The mold had the same dimensions of the spectrophotometer tip from one side.
5. Base line (T0) colorimetric analysis was carried out on all teeth by means of a spectrophotometer (Vita easy Shade ®spectrophotometer, Model# DEASY CHP, CBU, H013015. USA), according to the Commission Internationale de L’Eclairage (CIE) L*a*b* system (Fig. 3)
6. Before inducing caries lesion, the working area under testing was demarcated by a 4 mm × 4 mm white sticker representing the area of the acid attack. The rest of the tooth was covered by a black matte acid-resistant nail varnish.
7. All teeth were then immersed in a solution that contains 200 ml artificial caries solution (2.2 mmol/l KH_2_PO_4_, 2.2 mmol/l CaCl_2_, 50 mmol/l acetic acid) at pH 5 for a whole day [36]. The solution was changed daily till the appearance of the frosty white appearance.
8. The extent of white spot lesion was evaluated using a DIAGNOdent™ (KaVo, Bibberach, Germany) every day. Teeth were immersed again in an artificial caries solution if the extent of the lesion was not enough.
9. According to the readings of the DIAGNOdent™ [38] teeth were divided into four main groups 24 teeth each. The teeth were numbered and labeled. It took 15 days to produce the first white spot lesion.
  The readings of induced WSLs in the outer and inner half of the enamel ranged between 10 and 25.
  The readings of induced WSLs present in the outer and middle of the dentine without any signs of cavitation ranged between 26 and 37.
10. The color of the teeth in each group was spectrophotometrically measured after induction of the white spot lesions (T1).
11. Each of the main groups was subdivided into four groups, six teeth each according to the resin infiltration protocol used:
  - 1 etching + 1 infiltrant application (EA).
  - 1 etching + 2 infiltrant applications (EAA).
  - 2 etchings + 1 infiltrant application (EEA).
  - 2 etchings + 2 infiltrant applications (EEAA).

12. ICON® (DMG, Hamburg, Germany) resin infiltration was applied on the induced white spot lesions according to the manufacturer’s instructions:
  - 15% HCL (ICON-Etch) was applied for 2 min, then rinsed off with water spray for 30 s and dried. The number of etching applications was determined according to the group assigned.
  - Ethanol (ICON-Dry) was applied for 30 s followed by air-drying.
  - ICON-Infiltrant was applied and left on the tooth surface for 3 min. Excess resin was wiped away by a cotton roll and then light-cured for 40 s. In the groups with double resin infiltrant application, the application of infiltrant resin was repeated for 1 min and then light-cured for 40 s. Finally, the roughened enamel surface was polished using composite resin polishing discs and polishing cups.

13. The color of the teeth in each group was again spectrophotometrically measured after application of ICON® (T2).
  The steps of the study are illustrated in Fig. 4.

### Statistical analysis

To reduce the measuring error, two readings per tooth were taken for each time point. The reading was verified when the color difference between the readings obtained did not exceed the threshold of 1 ΔE unit. Readings with a difference of ΔE more than 1 unit were rejected and new readings were taken. An average of the two measurements was used when a difference of ΔE more than 1 unit persisted.

The data was collected and entered into the personal computer. Statistical analysis was done using Statistical Package for Social Science Version 20 (SSPS Inc., Chicago, III) software. After the data was tested for normality, it was decided to use one sample *t* test to compare the mean color difference ΔE to the threshold of clinical detection ΔE = 3.7. In order to compare the mean ΔE between the groups, one-way analysis of variance (ANOVA) was performed followed by post hoc test. The significance level was set at *P* < 0.05.

## Results

### Color change comparisons after WSL formation

Table 1 shows the mean color difference ΔE between the T0 (baseline) and T1 (after inducing the WSLs) in shallow enamel, deep enamel, shallow dentine, and deep dentine (3.84 ± 0.81, 4.81 ± 1.59, 5.11 ± 1.68, and 6.92 ± 0.86, respectively). The shallow enamel group did not show statistical difference when compared with the clinically detectable threshold (ΔE = 3.7); however, the deep enamel and shallow dentine groups showed significant difference (*P* < 0.05), while the deep dentine group showed high significant difference (*P* < 0.001).

**Table 1:** Comparison of the mean ΔE after WSL formation (T1-T0) between each main group and the threshold of clinical detection

Different depths of WSLs	Mean ΔE	SD	Critical value ΔE	*t*	*P* value
Shallow enamel (*n* = 24)	3.837	0.814	3.7	0.56	0.589
Deep enamel (*n* = 24)	4.807	1.586		2.31	0.043*
Shallow dentine (*n* = 24)	5.109	1.683		2.78	0.020*
Deep dentine (*n* = 24)	6.919	0.86		12.41	0.000**

*Significant at a *P* value < 0.05; **significant at a *P* value < 0.001; (*n*) sample size

**Fig. 5:**
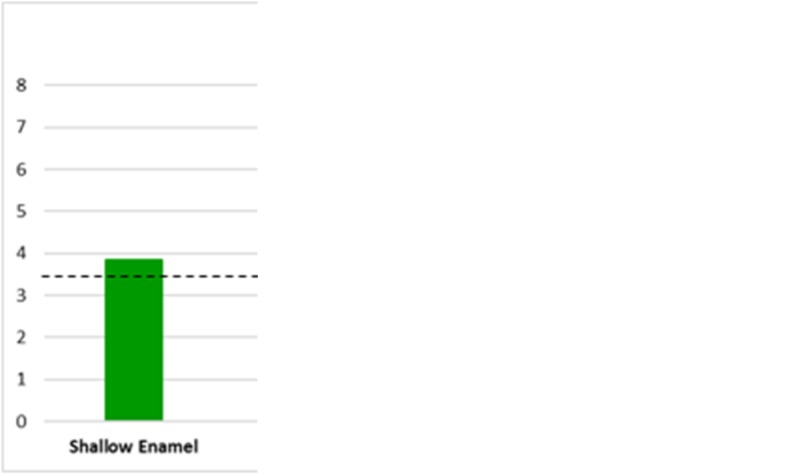
Bar graph showing the extent of the mean ΔE after WSL formation (T1-T0) in shallow enamel, deep enamel, shallow dentine, and deep dentine

There was clinically detectable WSLs in all of the groups, as all of them exceeded the threshold of clinical detection ΔE = 3.7 as shown in Fig. 5.

### Color change comparisons following the various protocols of resin infiltration

Table 2 displays the mean color difference ΔE between the baseline T0 and T2 following various resin infiltration protocols in shallow enamel. EA gave the least mean value 1.62 ± 0.85 followed by EAA, EEA and EEAA with means of 2.40 ± 0.63, 2.47 ± 0.8 and 2.99 ± 1.33, respectively. All groups were clinically not detectable as they did not exceed the clinically detectable threshold of ΔE = 3.7. All treatments demonstrated high significant difference (*P* < 0.001), when compared with the critical value, except for EEAA.

**Table 2:** Comparison between the mean ΔE (T2-T0) following various resin infiltration protocols of the WSLs in enamel and the threshold of clinical detection

Shallow enamel (*n* = 24)		Mean ΔE	SD	Critical value ΔE	*t*	*P* value
Resin infiltration protocols	EA (*n* = 6)	1.619	0.848	3.7	− 8.14	0.000**
	EAA (*n* = 6)	2.404	0.625		− 6.88	0.000**
	EEA (*n* = 6)	2.472	0.798		− 5.10	0.000**
	EEAA (*n* = 6)	2.993	1.334		− 1.76	0.109
Deep enamel (*n* = 24)						
Resin infiltration protocols	EA (*n* = 6)	2.418	0.308		− 13.80	0.000**
	EAA (*n* = 6)	1.952	0.397		− 14.60	0.000**
	EEA (*n* = 6)	2.979	0.814		− 2.94	0.015*
	EEAA (*n* = 6)	3.146	0.931		− 1.97	0.077

*Significant at a *P* value < 0.05; **significant at a *P* value < 0.001; (*n*) sample size

In addition, Table 2 illustrates the mean color difference ΔE between the baseline T0 and T2 following the different resin infiltration protocols in deep enamel. EAA gave the least mean value of 1.95 ± 0.4 followed by EA, EEA, and EEAA with means of 2.41 ± 0.31, 2.98 ± 0.81, and 3.15 ± 0.93, respectively. All groups were clinically not detectable as they did not exceed the clinically detectable threshold of ΔE = 3.7. EA and EAA showed high significant difference (*P* < 0.001), EEA showed significant difference (*P* < 0.05), and lastly, EEAA revealed no significant difference, when compared with the clinically detectable threshold.

Table 3 presents the mean color difference ΔE between the baseline T0 and T2 following the different resin infiltration protocols in shallow dentine. The least mean value 3.0 ± 0.45 was observed with EAA followed by EA, EEA, and EEAA with means of 3.55 ± 0.32, 3.83 ± 0.36, and 4.19 ± 0.34, respectively. Only two groups were clinically detectable (EEA, EEAA) as they exceeded the clinically detectable threshold of ΔE = 3.7. EEAA showed significant difference (*P* < 0.05) and EAA revealed high significant difference (*P* < 0.001), when compared with the critical value ΔE = 3.7.

**Table 3:** Comparison between the mean ΔE (T2-T0) following various resin infiltration protocols of the WSLs in dentine and the threshold of clinical detection

Shallow dentine (*n* = 24)		Mean ΔE	SD	Critical value ΔE	*t*	*P* value
Resin infiltration protocols	EA (*n* = 6)	3.552	0.317	3.7	− 1.55	0.153
	EAA (*n* = 6)	3.002	0.447		− 5.18	0.000**
	EEA (*n* = 6)	3.826	0.361		1.16	0.274
	EEAA (*n* = 6)	4.190	0.340		4.78	0.001*
Deep dentine (*n* = 24)						
Resin infiltration protocols	EA (*n* = 6)	4.029	0.537		2.03	0.070
	EAA (*n* = 6)	3.763	0.598		0.35	0.734
	EEA (*n* = 6)	4.732	0.773		4.43	0.001*
	EEAA (*n* = 6)	5.964	0.567		13.24	0.000**

*Significant at a *P* value < 0.05; **significant at a *P* value < 0.001; (*n*) sample size

Table 3 also demonstrates the mean color difference ΔE between the baseline T0 and T2 following various resin infiltration modalities in deep dentine. EAA had the least mean value of 3.76 ± 0.6, followed by EA, EEA, and EEAA with means of 4.03 ± 0.54, 4.73 ± 0.77, and 5.96 ± 0.57, respectively. All groups displayed clinical detection, i.e., exceeded ΔE = 3.7. EEA showed significant difference (*P* < 0.05) and EEAA showed high significant difference (*P* < 0.001), when compared with the clinical detectable threshold.

Table 4 shows multiple comparisons of the mean color difference ∆E (T2-T0) between the different resin infiltration protocols in shallow enamel. It was found that EA significantly differ from the other groups: highly significant (*P* < 0.001) when compared with EEAA with a mean difference of 1.374 and significantly different (*P* < 0.05) when compared with EAA and EEA with mean differences of 0.785 and 0.853, respectively. Those mean differences were obtained from subtraction of the respective means presented in Table 2. When comparing the other groups with each other, there was no significant difference.

**Table 4:** Multiple comparisons for the mean ΔE (T2-T0) between the different resin infiltration protocols in the shallow and deep enamel groups

Shallow enamel	EAA	EEA	EEAA
EA	0.013*	0.006*	0.000**
EAA		0.992	0.091
EEA			0.160
Deep enamel	EAA	EEA	EEAA
EA	0.045*	0.009*	0.001*
EAA		0.000**	0.000**
EEA			0.763

*Significant at a *P* value < 0.05; **significant at a *P* value < 0.001

While in deep enamel, it was found that all the comparisons were significant except for EEA with EEAA. EAA gave high significant difference (*P* < 0.001) when compared with EEA and EEAA with mean differences of 1.027 and 1.194, respectively. Upon comparing EA with the rest of the modalities, significant difference (*P* < 0.05) was found between them.

Table 5 shows multiple comparisons of the mean color difference ∆E (T2-T0) between the different resin infiltration protocols in shallow dentine. It was found that all the comparisons are significant except for EEA with EEAA. High significant difference (*P* < 0.001) was found when comparing EA with EEA and EEAA with mean differences of 0.274 and 0.638, respectively. Also, high significant difference (*P* < 0.001) was found when comparing EAA with EEA and EEAA with mean differences of 0.824 and 1.188, respectively.

**Table 5:** Multiple comparisons for the mean ΔE (T2-T0) between the different resin infiltration protocols in the shallow and deep dentine groups

Shallow dentine	EAA	EEA	EEAA
EA	0.008*	0.000**	0.000**
EAA		0.000**	0.000**
EEA			0.107
Deep dentine	EAA	EEA	EEAA
EA	0.019*	0.000**	0.000**
EAA		0.002*	0.000**
EEA			0.000**

*Significant at a *P* value < 0.05; **significant at a *P* value < 0.001

**Fig. 6:**
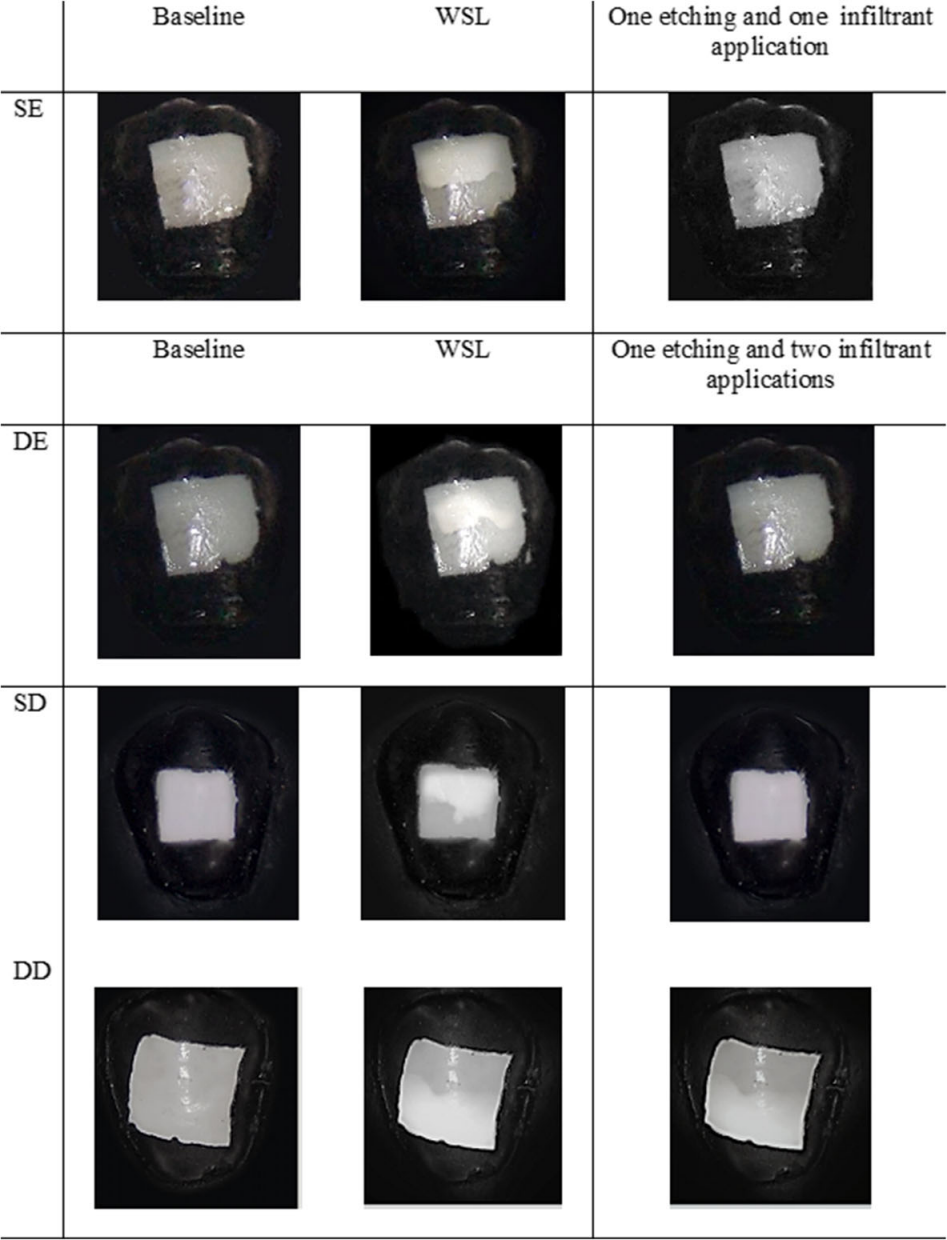
The best resin infiltration protocols in the different depths of WSLs. SE shallow enamel, DE deep enamel, SD shallow dentine, DD deep dentine

Whereas in deep dentine, it was found that all the resin infiltration protocols show significant difference when compared with each other. When comparing EEAA with EA, EAA, and EEA, there was a high significant difference (*P* < 0.001) with mean differences of 1.935, 2.201, and 1.232, respectively. Also, high significant difference (*P* < 0.001) was found upon comparing EA with EEA with mean difference of 0.703. Furthermore, significant difference (*P* < 0.05) is shown when comparing EA with EAA with a mean difference of 0.266 and when comparing EAA with EEA with a mean difference of 0.969. The best resin infiltration protocols in the different depths of WSLs are demonstrated in Fig. 6.

## Discussion

Results in this study showed that there was a difference between the initial color of the tooth and its color after the induction of the WSL. The deeper the WSL, the more clinically apparent it was. Also, as mean ∆E exceeded 3.7 units, the lesion got deeper. Shallow enamel lesions were clinically detectable although they were insignificant when compared with the critical value. When the four groups were compared spectrophotometrically, deep dentine showed the highest color difference.

A previous study by Gray and Shellis [26] showed increased penetration depth with increased number of etching; however, the depths of the lesion were not taken in consideration. Knösel and colleagues [27] recommended more etching in long-standing deep WSLs. Moreover, Arnold et al. [28] concluded that repeated conditioning with 15% hydrochloric acid increased the depth of the etched surface erosion. Therefore, in the current study, eight groups were assigned for double etching with 15% HCL for 120 s each, to evaluate the effect of increased etching prior to resin infiltration, on the color change of different depths of WSLs.

Also, eight groups were assigned for double infiltrant application: 3 min for the first step and further 1 min for the second step according to manufacturer’s instructions. This was suggested by two studies [24, 33] which noticed decreased lesion pore volume after double resin infiltrant application. Also, the resin infiltrant was recommended to be applied twice by Robinson et al. [39] because of the shrinkage of the material following the first application resulting in the generation of space that can then be occluded by a second application.

Two studies by Kim et al. [32] and Ou et al. [33] examined the association between the depth of WSLs and the effect of resin infiltration, but no evidence was given about the number of etching and infiltrant applications needed for each depth.

The main goal of treatment of the WSL is to arrest the progression of non-cavitated lesions and to improve the esthetics by diminishing their opacity. Starting with lesions in the outer enamel, the depth of the WSLs was shallow and one etching with one infiltrant application gave the least mean color difference when compared to the baseline color, providing the best result, as it nearly completely camouflaged the WSL. Therefore, it is advisable to use single etching with one infiltrant application to save time and material. However, double infiltrant application with double etching gave the highest mean color difference, statistically insignificant when compared with the threshold of clinical detection. This might be related to surface roughness produced by the resin. It was postulated that the average value of the resin color difference was related to the surface roughness, which might be decreased by polishing [4, 40, 41].

Similarly, in deep enamel, the results showed that various resin infiltration protocols displayed no clinical detection of the WSLs as they did not exceed the clinically detectable threshold; the WSLs were well masked. The least mean color difference was noticed with one etching and double infiltration modality when compared to the initial teeth color. When comparing the four modalities, double etching with double infiltrant application improved the color but not efficiently, as the mean ∆E approached the clinical detectable threshold. This might be due to the double etching step that could have led to differences in enamel prism morphology. This was proved previously by Arnold et al. [28] but not in accordance to a certain depth.

However, going deeper to the lesions reaching shallow dentine where the WSLs were deeper than the previous two groups of enamel lesions, larger differences in color were found when compared with teeth color at baseline. Only two groups gave the best color match to the baseline; one etching step with either one or two infiltrant applications, their values did not exceed the clinical detectable threshold of ∆E. This might suggest that one etching step is preferable for lesions reaching the outer layers of dentine regardless of the number of infiltrant applications that will be done. To be more precise when four groups were compared, one etching with two infiltrant applications camouflaged the WSL giving the least mean value implicating the best combination of treatment for this depth of WSL. On the other hand, double etching with double infiltrant applications gave a mean value that exceeded the critical value of ∆E, presenting the worst treatment modality.

Progressing in deep dentine lesions, the WSLs were the deepest reached in this study. All the treatment modalities showed clinical detection after resin infiltration, i.e., different resin infiltration protocols were not successful in completely masking the WSLs. Furthermore, the worst result was found in double etching with double infiltrant application and the best was found in one etching with a double infiltrant application. One etching was found to be enough to open the lesion pores without weakening of the tooth, while double etching resulted in exfoliation of enamel surface in some of the samples. This finding was in accordance with that of Hammad et al. [42] who postulated that removal of the hypermineralized surface layer by 15% HCL might additionally weaken the lesion structure. Thus, in deep dentine WSLs, one etching was found to be enough to remove the hypermineralized layer without losing any enamel tissues besides improving the color.

It is preferable in deep dentine lesions to first try using the infiltration resin technique in an attempt to improve the color. However, according to the results of this study, we cannot anticipate the full masking of the lesions once it reach the deep layers of the dentine. If the gained result was not satisfactory and the WSL was still clinically visible, unfortunately, the treatment mean will be diverted to more invasive restorative procedures.

## Conclusions

Based on the results of the current study, it can be concluded that:

1. As the WSL got deeper, the color of the lesion became more opaque and clinically visible.
2. Resin infiltration concept is a good technique for its chameleon effect used for different depths of WSLs.
3. With shallow enamel, the best treatment option is to perform one etching step with one application of resin infiltrant.
4. For deep enamel and shallow dentine, the best treatment modality is one etching step and two applications of the resin infiltrant.
5. In deep dentine without cavitation, lesions were partially masked in all the treatment modalities and remained clinically detectable.
6. It is not advisable to perform two etching steps as enamel exfoliation, in some of the samples, was observed especially in deeper dentine lesions.

## Abbreviations

CIE: Commission Internationale de l’Eclairage
DD: Deep dentine
DE: Deep enamel
EA: 1 etching + 1 application
EAA: 1 etching + 2 applications
EEA: 2 etchings + 1 application
EEAA: 2 etchings + 2 applications
HCL: Hydrochloric acid
ICON: Infiltration concept
RI: Resin infiltration
SD: Shallow dentine
SE: Shallow enamel
TEGDMA: Tetraethylene glycol dimethacrylate
WSL: White spot lesions
